# Rapid, controllable growth of silver nanostructured surface-enhanced Raman scattering substrates for red blood cell detection

**DOI:** 10.1038/srep24503

**Published:** 2016-04-20

**Authors:** Shu Zhang, Xueli Tian, Jun Yin, Yu Liu, Zhanmin Dong, Jia-Lin Sun, Wanyun Ma

**Affiliations:** 1 College of Science, Huazhong Agricultural University, 430070, Wuhan, China; 2 State Key Laboratory of Low-Dimensional Quantum Physics, Department of Physics, Tsinghua University, 100084, Beijing, China; 3 Collaborative Innovation Center of Quantum Matter, Beijing, China

## Abstract

Silver nanostructured films suitable for use as surface-enhanced Raman scattering (SERS) substrates are prepared in just 2 hours by the solid-state ionics method. By changing the intensity of the external direct current, we can readily control the surface morphology and growth rate of the silver nanostructured films. A detailed investigation of the surface enhancement of the silver nanostructured films using Rhodamine 6G (R6G) as a molecular probe revealed that the enhancement factor of the films was up to 10^11^. We used the silver nanostructured films as substrates in SERS detection of human red blood cells (RBCs). The SERS spectra of RBCs on the silver nanostructured film could be clearly detected at a laser power of just 0.05 mW. Comparison of the SERS spectra of RBCs obtained from younger and older donors showed that the SERS spectra depended on donor age. A greater proportion of the haemoglobin in the RBCs of older donors was in the deoxygenated state than that of the younger donors. This implies that haemoglobin of older people has lower oxygen-carrying capacity than that of younger people. Overall, the fabricated silver substrates show promise in biomedical SERS spectral detection.

Considerable theoretical and experimental research has shown that surface plasmon resonance readily occurs on the surface of noble metal nanostructures excited by incident photons and results in marked local field enhancement^1^,^2^,^3^,^4^,^5^,^6^,^7^. Therefore, noble metal nanomaterials are widely used in surface-enhanced Raman scattering (SERS), which is a powerful tool to study molecular vibration spectra and for chemical analysis^3^,^4^. Silver is often used for SERS substrates because of the cost of gold. So researchers have focused on the preparation methods of silver nanomaterials and the improvement of their SERS performance.

Various methods to prepare nanomaterials have been reported; common examples include magnetron sputtering^8^, thermal evaporation^9^, template-based methods^7^,^10^,^11^,^12^,^13^, and nanocrystal catalysis^14^,^15^,^16^. Besides, the solid-state ionics method improved by our research group also has the advantages of convenience and controllability^17^,^18^. This method does not use templates so template removal or sample purification is not required and silver nanostructures are grown in the solid phase and can conveniently be directly picked up by tweezers. However, this method originally took weeks to grow centimetre-scale nanomaterials because the external direct current was only 3–8 μA^17^,^18^,^19^,^20^. Our experiments improved the growth rate of nanomaterials by increasing the intensity of the external direct current, meaning we are now able to obtain centimetre-scale nanomaterials in just hours. In this study, we focus on the surface morphology and SERS properties of silver nanostructures grown with an external direct current of tens of microamps and explore the ability of the solid-state ionics method to quickly grow controlled silver nanostructures that are promising for biomedical detection.

Qualitative and quantitative chemical analyses of human blood are widely used in routine examinations or diagnostics for a wide variety of diseases due to its easy access and key roles in biological activity. Measurement of red blood cells (RBCs), which are an important component of blood, allows routine monitoring of many pathologies and the general health of individuals. Raman spectroscopy is an attractive tool to measure RBCs because it requires no extrinsic labelling and is not limited by the presence of water. In recent years, the study of RBCs by Raman spectroscopy has gradually increased^21^. RBCs strongly absorb 514 nm excitation light in resonance Raman spectroscopy, which lead to cell damage. Thus, researchers usually excite RBCs at 785 nm using a laser power of about ~5 mW, which is relatively high^21^,^22^,^23^. However excitation of RBCs at 514 nm provides obvious bands in the central region (1200–1400 cm^−1^) that are reported to be sensitive to the oxidation and spin state of the central metal atom within the porphyrin macrocycle^22^,^23^,^24^. Using SERS substrates, the intensity of the Raman spectrum of RBCs can be enhanced substantially so that the laser power can be decreased to a low level while the Raman spectral information remains intact. In this study, we use the silver nanostructured substrates rapidly grown by the solid-state ionics method in SERS detection of RBCs.

## Results

### The surface enhancement ability of silver nanostructures grown at different external direct current

**Figure 1:**
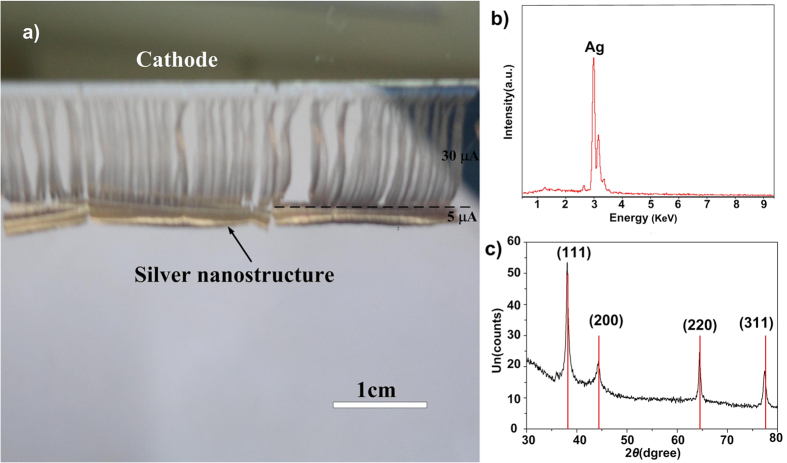
Characterization of silver nanostructures grown by the solid-state ionics method. (**a**) Camera image of the silver nanostructure. (the ~2 mm length denser region below dash line is grown with 5 μA for 6 h; the ~1 cm length sparser region above dash line is grown with 30 μA for 2 h. (**b**) X-ray EDS and (**c**) XRD pattern of the silver nanostructure grown with an external current of 30 μA for 2 h.

Characterization data for the silver nanostructures grown with an external current of 30 μA for 2 h by the solid-state ionics method are presented in Fig. 1a. The nanostructures cover an area of about 5 cm × 1 cm, which is very rapid growth. In fact, we found that the growth rate of silver nanostructures is proportional to the intensity of the external direct current^18^. X-ray energy-dispersive spectroscopy (EDS) results (Fig. 1b) showed that the silver nanostructured film is pure silver. The X-ray diffraction (XRD) pattern (Fig. 1c) contained a high-intensity diffraction peak at about 38.20° and other peaks were observed at about 44.42°, 64.40°, and 77.32° corresponding to the (111), (200), (220), and (311) crystalline planes of the cubic crystalline structure of Ag (JCPDS card file no. 4783), respectively.

**Figure 2:**
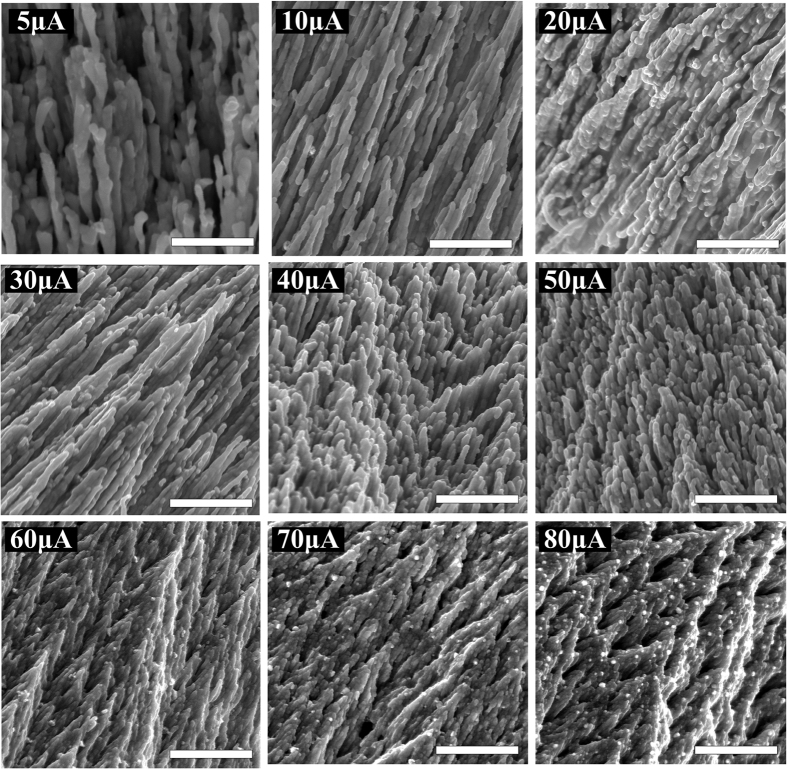
SEM images of the front surface morphology of the silver nanostructures grown with different external direct currents. The scale bar is 1 μm in all images.

Scanning electron microscopy (SEM) images of silver nanostructures grown with different external direct currents are presented in Fig. 2. The surface morphology of the silver nanostructures depends on the external direct current. This is because the surface morphology is influenced by the accumulation rate of Ag atoms at the cathode. When the external direct current is low, the current density of Ag^+^ in the RbAg_4_I_5_ film used as an ionic conductor is small and the accumulation rate of Ag atoms at the cathode edge is slow. As a result, the Ag atoms accumulate in an orderly manner to form thin, ordered nanowires. At higher external direct current, the accumulation rate of Ag atoms at the cathode edge is faster, which leads to thicker Ag nanowires. Redundant Ag atoms continue to accumulate on the Ag nanowires to form convex particles. When the external direct current exceeds 30 μA, the accumulation rate of Ag atoms at the cathode edge increases further. Nanowires cannot adapt to such a high accumulation rate, so closely stacked nanobud structures are produced. The density of the nanobuds increases with external direct current up to 70 μA. When the external direct current exceeds 70 μA, the nanobuds fuse to form thicker structures coated with numerous convex particles.

**Figure 3:**
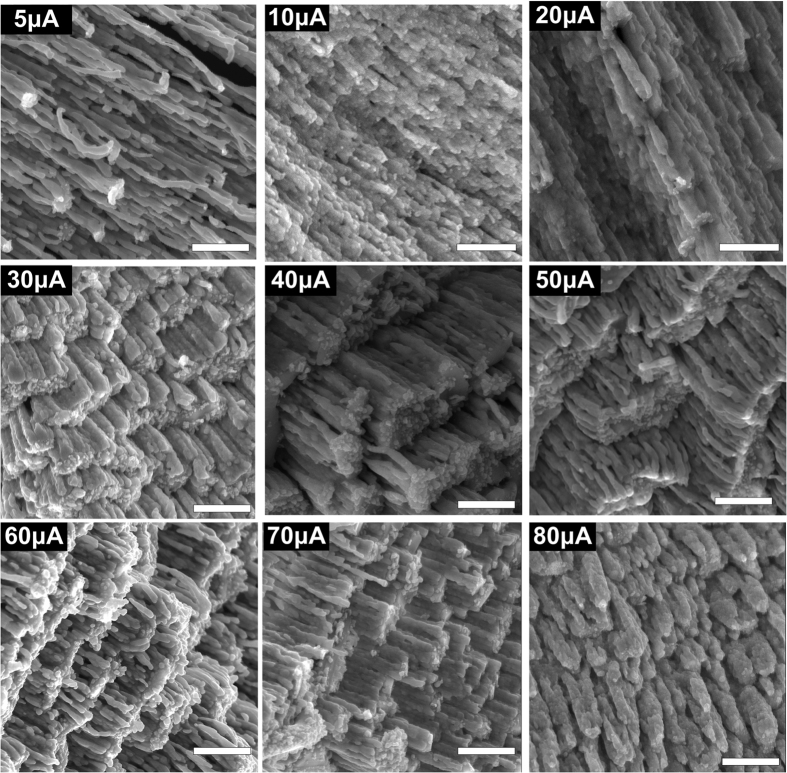
SEM images of the morphology of the back of silver nanostructures grown with different external direct currents. The scale bar is 1 μm in all images.

The morphology of the back of the silver nanostructures was also characterized by SEM (Fig. 3). These images clearly show how the morphology of the back of the silver nanostructures changes with external direct current. When the current is lower than 10 μA, the morphology of the back of the nanostructures is still ordered nanowires, which is similar to the morphology on the front of the sample. When the current is higher than 20 μA, the back of the silver nanostructures resembles a stair-like structure composed of silver nanoclusters. The clusters are the roots of nanostructures, and their tips are present at the front surface as nanobuds. As external direct current increases, the stairs become denser. Thus, the silver nanobuds on the front surface also become denser, which will lead to more “hot spots” and better surface enhancement. However, when the external direct current was too high (80 μA), the back surface consisted of thick nanorods that provided fewer “hot spots” on the front surface than the nanobuds.

**Figure 4:**
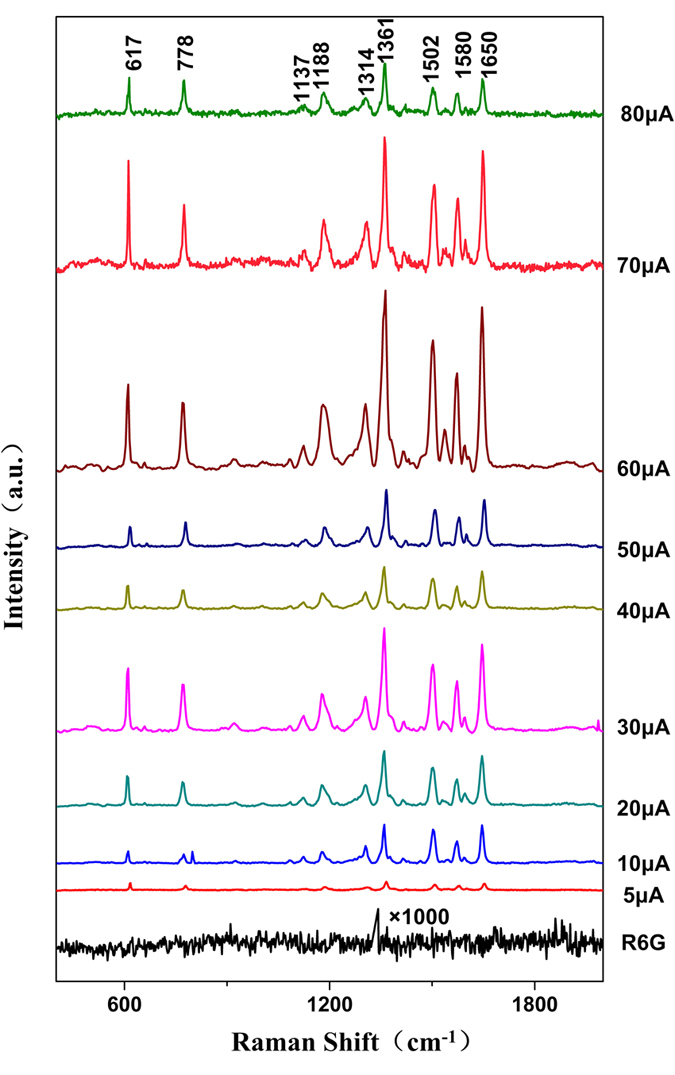
SERS spectra for 10^−6 ^mol L^−1 ^R6G on silver nanostructure substrates grown with different external currents. The spectrum labelled R6G is that of R6G on a glass slide as control.

R6G was used to examine the enhancement effect of the silver nanostructures as SERS substrates. The results are provided in Fig. 4. The intensity of the SERS spectra of R6G appears rising trend with growth current for the ranges of 5–30 μA and 40–60 μA. At 60 μA the intensity of SERS spectrum reaches highest. When growth current exceeds 60 μA, the intensity of SERS spectra decreased.

The enhancement factor (E_F_) of the samples grown at 60 μA was calculated by:


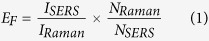


where *I*_*SERS*_ is the SERS spectral intensity of R6G on a SERS substrate, N_*SERS*_ is the number of R6G molecules probed on the SERS substrate; *I*_*Raman*_ is the Raman spectral intensity of R6G without SERS substrate, *N*_*Raman*_ is the number of R6G molecules probed without SERS substrate. Details of the calculation were provided in Supplementary Information.

For substrates grown at 60 μA, E_F_ was 10^11^. This demonstrates that the nanostructures prepared by the solid-state ionics method using an external direct current of tens of microamps are suitable as SERS substrates. Because the preparation of such substrates is convenient, rapid and controllable, these substrates are promising for use in biological applications.

### SERS spectra detection of RBCs using silver nanostructured substrates

**Figure 5:**
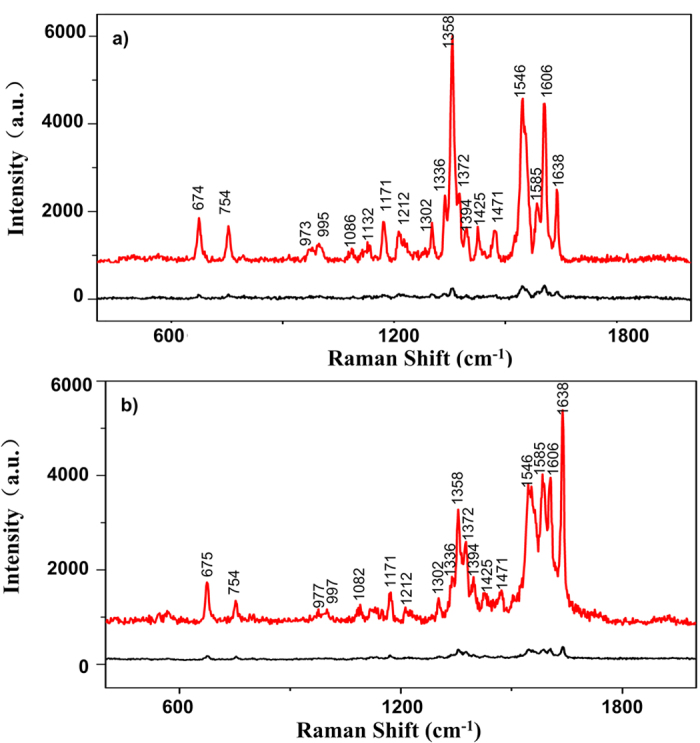
Two categories of SERS spectra of RBCs on the SERS substrate. In (**a**), intensity of 1358 cm^−1^ is much higher than that of other characteristic peaks; In (**b**), intensity of 1638 cm^−1^ is much higher than that of other characteristic peaks. For both (**a**) and (**b**), the red curves are the SERS spectra of RBC on SERS substrate while the black curves are the Raman spectra of RBC on glass slide.

We used the silver nanostructures grown with an external current of 60 μA as SERS substrates for RBCs according to the results in Fig. 4. Since this substrate has marked Raman enhancement, we were able to use a low laser power of just 0.05 mW, which minimized the damage to samples without losing spectral details. Figure 5 shows two categories of SERS spectra of RBCs on SERS substrates. In Fig. 5a the intensity of 1358 cm^−1^ is much higher than that of other characteristic peaks; In Fig. 5b the intensity of 1638 cm^−1^ is much higher than that of other characteristic peaks. Raman spectra of RBCs on glass slides were measured under the same conditions as controls. On the SERS substrates, the intensity of the spectrum of RBCs is more than one order of magnitude higher than that on glass slides. Moreover, the SERS spectra of RBCs on SERS substrates contain more peaks and details than the control spectra. In contrast, the peaks in this region of the spectra obtained for RBCs on glass slides are almost masked by noise and difficult to detect. Therefore, our silver nanostructured SERS substrates allow SERS spectral detection of RBCs by 514 nm excitation at low laser power.

Haemoglobin is a globular protein that facilitates oxygen transport by binding to heme groups, and the main component of the cytoplasm of RBCs. Using a two-state model, the protein is generalised to alternate between two structures: a deoxygenated, low-affinity, tense (T) structure and an oxygenated, high-affinity, relaxed (R) structure^22^,^23^,^24^,^25^. All of the bands observed for the RBCs samples (shown in Fig. 5) can be assigned to normal modes of either oxygenated or deoxygenated haemoglobin, as shown in Supplementary Table S1.

Characteristic peaks of the T state are different from those of the R state. The T state of haemoglobin (deoxygenated) contains peaks at 1358 and 1606 cm^−1^, while the R state of haemoglobin (oxygenated) has peaks at 1372 and 1638 cm^−1^. The relative enhancement of the characteristic peaks of R6G in SERS spectra (i.e. the enhancement of different characteristic peaks in one SERS spectrum) is consistent for different substrates or different positions on same substrate grown with 60 μA (Figs 1S and 2S in the Supplementary Information). Therefore we believe that for each SERS spectrum of hemoglobin, the relative enhancement of the above four characteristic peaks is consistent. For each spectrum, we compared the intensity ratios between peak 1358 cm^−1^ and 1372 cm^−1^, which is denoted as I_1358_/I_1372_ and compared the intensity ratios between peak 1606 cm^−1^ and 1638 cm^−1^, which is denoted as I_1606_/I_1638_. When I_1358_/I_1372_ and I_1606_/I_1638_ are higher, more of the haemoglobin in the RBCs is in the deoxygenated T state. Conversely, when I_1358_/I_1372_ and I_1606_/I_1638_ are lower, more of the haemoglobin in the RBCs is in the oxygenated R state. The RBC samples were separated into two groups depending on donor age: Group 1 (60–85 years old) and Group 2 (18–30 years old).

**Figure 6:**
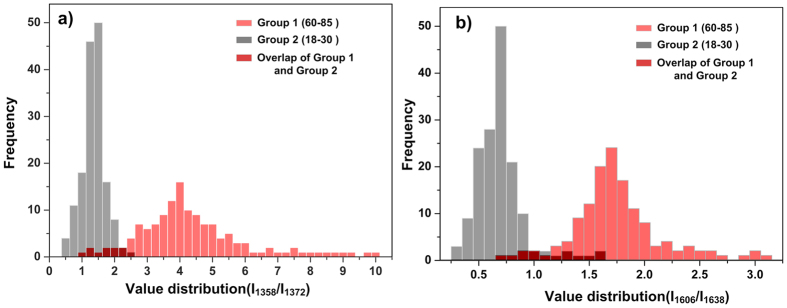
Detection of the oxygenation of haemoglobin using SERS spectroscopy. (**a**) Distribution of value of I_1358_/I_1372_ for both groups. (**b**) Distribution of value of I_1606_/I_1638_ for both groups. The light red histogram represented Group 1while the grey histogram represented Group 2. The dark red part is the overlap of both groups. The SERS spectra number brought into statistics of Group 1 is n = 138, and that of Group 2 is n = 156.

Figure 6 presents the statistical distributions of I_1358_/I_1372_ and I_1606_/I_1638_ for Group 1 (age range of 60–85) and Group 2 (age range of 18–30). Figure 6a showed that the value of I_1358_/I_1372_ of the RBCs of Group 1 is mainly distributed in the range of 2.5–6 with the maximum in the range of 3.75–4.25. Meanwhile the value of I_1358_/I_1372_ of the RBCs of Group 2 is mainly distributed in the range of 0.5–2 with the maximum in the range of 1.25–1.5. In Fig. 6b, it showed the distribution of the value of I_1606_/I_1638_ of Group 1 and Group 2. For RBCs of Group 1, the value of I_1606_/I_1638_ is mainly distributed in the range of 1.4–2.0 with the maximum in the range of 1.6–1.7. For RBCs of Group 2, the value of I_1606_/I_1638_ is mainly distributed in the range of 0.3–1 with the maximum in the range of 0.6–0.75. As discussed above, these results indicate that the haemoglobin in RBCs obtained from donors in Group 1 tends to be in the T state more than that of the haemoglobin of RBCs isolated from donors in Group 2, which reveals that the level of haemoglobin oxygenation in Group 1 is lower than that in Group 2. Donors in Group 1 are much older than those in Group 2. Therefore, it seems likely that the observed difference of the haemoglobin state between the two groups is because the degree of activity and metabolism of the people in Group 1 will be much lower than those of the people in Group 2. Moreover, older people are more likely to have underlying disease than younger ones, which may also influence the level of haemoglobin oxygenation in their blood samples.

## Discussion

This study focuses on rapid, controllable growth of centimetre-scale silver nanostructured substrates prepared by solid-state ionics method and the application in SERS spectra detection of RBCs. Silver nanostructured substrates with various surface morphologies were prepared using an external direct current of tens of microamps, which is able to prepare centimetre-scale silver nanostructure films in hours rather than weeks. The morphology of substrates depended on the external direct current. The intensity of the SERS spectrum increased with growth current from 5–30 μA and 40–60 μA. The maximum intensity of the SERS spectrum was reached at 60 μA. When growth current exceeded 60 μA, the intensity of SERS spectra decreased. The enhancement factor of the substrates grown at 60 μA can up to 10^11^, which is remarkable.

The substrates showed good performance in the SERS spectral detection of RBCs. With newly made substrates, detailed SERS spectra of RBCs can still be detected at a very low laser power of 0.05 mW, ensuring there is no RBC damage and no spectral information loss. The difference of haemoglobin state between younger and older donors was clearly obtained. The level of haemoglobin oxygenation in the older donors group was lower than that in the younger donors group. This may be because the degree of activity and metabolism of the older donors are much lower than that of the younger donors and older people are more likely to have underlying disease than younger ones, which may also influence the level of haemoglobin oxygenation in their blood samples. Although the detection was only carried using RBC samples from healthy donors in this work, the silver nanostructured substrates show the potential for RBC analysis and biological macromolecule detection. This work also laid a foundation for future detailed study of other age group (30–60 years, teenager, child etc.) and investigation of other blood components. Further studies are needed to develop silver substrates with surface modification methods which will make the substrates easier and longer to store and have higher biological compatibility.

## Experimental methods

### Preparation of silver nanostructures by the solid-state ionics method

**Figure 7:**
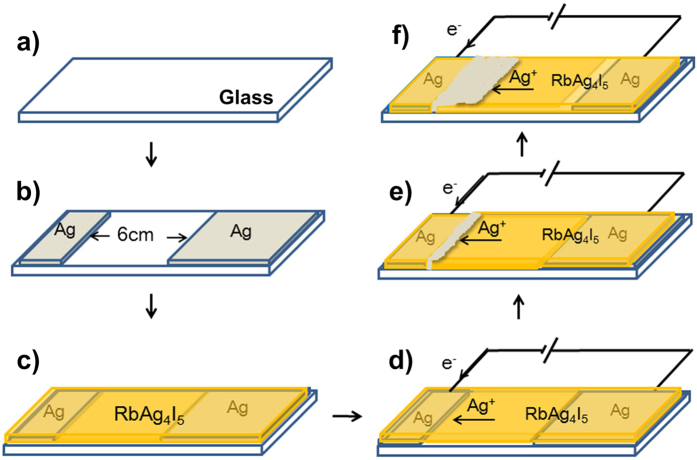
Growth of silver nanostructures by the solid-state ionics method. (**a**) A clean glass slide placed in a deposition chamber at room temperature under vacuum (10^−4^ Pa). (**b**) Two parallel silver films separated by a distance of 6 cm deposited by vacuum thermal evaporation on the two ends of the glass slide for use as electrodes. (**c**) RbAg_4_I_5_ film (400-nm-thick) deposited over the Ag electrodes and glass slide by vacuum thermal evaporation. (**d**) An external direct current applied to the slide. (**e**) Silver nanostructures grow at the edge of the cathode and burst out of the RbAg_4_I_5_ film. (**f**) The silver nanostructures grow continuously under the applied current. The growth front, where Ag^+^ is reduced to Ag atom, serves as the new edge of the cathode. The silver nanostructure grows toward the anode and the silver anode is consumed.

A superionic conducting RbAg_4_I_5_ film has the same order of ionic conductivity as a strong electrolyte solution or fused salt, so it acts as an ion channel between silver electrodes. With the external direct current provided by a source meter (Keithley 2400, USA), Ag atoms of the anode are ionized to form Ag^+^ and move to the cathode through the ion channel, thus forming a directional ion current. The Ag^+^ is reduced to Ag atom at the cathode. As a result, Ag atoms pile up at the edge of the cathode and grow into silver nanostructures. This process is described in detail in Fig. 7.

### Description of RBC sample donors

In this study, all the blood samples provided by the Hospital of Tsinghua University (Beijing, China) were collected by venipuncture from healthy donors following standard protocols. Details about the age and gender of each donor are listed in Supplementary Table S2. All methods involving human blood samples were performed according to national regulations concerning research on human biological samples. For all cases, oral informed consent is available. The ethics committee of Tsinghua University approved the study. The donors were anonymous to the investigators who participated in the study.

### RBC sample preparation

All RBC samples were put into single-use blood collection tubes containing EDTA as anticoagulant. The samples were divided into two different groups depending donor age. Group 1 (12 people) consisted of donors aged 60–85 years, and Group 2 (15 people) consisted of donors aged 18–30 years. We chose the donors’ age distribute in the <30 years old group and >60 years old group to expand the age difference of the donors’, which will easier to distinguish the difference of main characteristic peaks relative intensity of SERS spectra of RBC caused by age factor.

RBCs were separated from peripheral blood as follows:

1. Peripheral blood was diluted with 0.9% saline solution (1:1 v/v).
2. Lymphocyte separation medium (Solarbio Science and Technology Co., Ltd, 3 mL) was placed in a 15-ml centrifuge tube.
3. The diluted blood (3 mL) from step 1 was carefully overlaid on the liquid surface of the lymphocyte separation medium and the tube was centrifuged at 2200 rpm for 20 min.
4. Physiological saline solution (0.9%, 3 mL) was added to another 15-ml centrifuge tube.
5. The RBCs separated at the bottom of the tube after centrifugation in step 3 were added to the tube prepared in step 4 using a pipette.
6. The tube prepared in step 5 was centrifuged at 2000 rpm for 10 min. After centrifugation, the supernatant was discarded to give the isolated RBCs.

### SERS spectra detection

For SERS spectra detection of R6G, newly grown silver nanostructures were fixed on a clean slide as the SERS substrate, 10 μl of R6G solution (10^−6 ^mol L^−1^) was added onto the substrate. A clean 24 mm × 24 mm coverslip was placed on the slide to seal in the R6G solution. The detection was carried out using a confocal Raman spectrometer (LabRAM HR Evolution, Horiba Jobin Yvon, France). The excitation light was a focused Ar^+^ laser (excitation wavelength 514 nm, focal area ~2 μm × 2 μm with a 50× objective). The exposure time was 20 s and the detection range was 400–2000 cm^−1^. The power of the laser was 0.5 mW. Raman spectra measurements were carried out at room temperature.

For SERS spectra detection of RBCs, the conditions were the same as that used to detect R6G except for the laser power, which was decreased to 0.05 mW. A 10 μl drop of RBC sample prepared above was added onto the substrate, followed by placement of a clean 24 mm × 24 mm coverslip on the slide. For each sample, more than 10 cells were measured and statistical averages were calculated to ensure the accuracy of the results.

## Additional Information

**How to cite this article**: Zhang, S. *et al.* Rapid, controllable growth of silver nanostructured surface-enhanced Raman scattering substrates for red blood cell detection. *Sci. Rep.* **6**, 24503; doi: 10.1038/srep24503 (2016).

## Supplementary Material

Supplementary Information
