# Supplementary material for: Rapid, controllable growth of silver nanostructured surface-enhanced Raman scattering substrates for red blood cell detection
Source: Sci Rep. 2016 Apr 20;6:24503. doi: 10.1038/srep24503 (PMC4837339; doi:10.1038/srep24503)
Supplement: Supplementary Information [file srep24503-s1.doc]

**Supplementary Information**

**Rapid, controllable growth of silver nanostructured surface-enhanced Raman scattering substrates for red blood cell detection**

*Shu Zhang^1,2^, Xueli Tian^2^, Jun Yin^2^, Yu Liu^2^, Zhanmin Dong^2^, Jia-Lin Sun^2,3^, Wanyun Ma^2,3^*

^1^College of Science, Huazhong Agricultural University, 430070, Wuhan, China. ^2^State Key Laboratory of Low-Dimensional Quantum Physics, Department of Physics, Tsinghua University, 100084, Beijing, China. ^3^Collaborative Innovation Center of Quantum Matter, Beijing, China. Correspondence should be addressed to Jia-Lin Sun (email: [jlsun@tsinghua.edu.cn](mailto:jlsun@tsinghua.edu.cn)) or Wanyun Ma (email: mawy@tsinghua.edu.cn).

1. **Reproducibility of the SERS performance of the substrates**

We used 10^−10^ mol•L^−1^ R6G to examine the reproducibility of the SERS performance of the substrates. All detections were carried out under the same conditions using a confocal Raman spectrometer (LabRAM HR Evolution, Horiba Jobin Yvon, France). The excitation light was a focused Ar+ laser (excitation wavelength 514 nm, focal area ~2×2 μm with a 50× objective). The exposure time was 10 s and the detection range was 1000–1800 cm−1. The power of the laser was 0.5 mW.

We chose ten points randomly in the same substrate and measured their SERS spectral intensities. The results are shown in Fig. 1S. Fig. 1S reveals that the SERS spectral intensities at different points on the same substrate are quite similar. Fig. 2S displays the SERS spectral intensities of six different substrates produced with the same external current (60 μA). For different substrates produced in the same way, the SERS spectral intensities differ only slightly. These results demonstrate that the overall reproducibility of the SERS performance of the substrates is good enough to allow their use as substrates in further experiments.


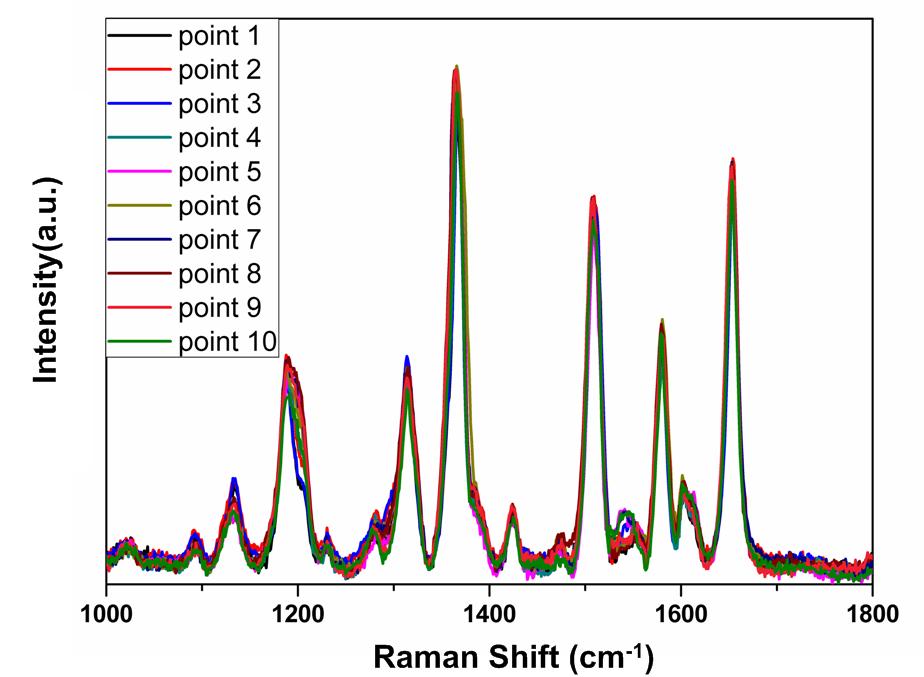


**Fig.1S** The SERS spectra intensity of 10 different points randomly distributed on same SERS substrate.


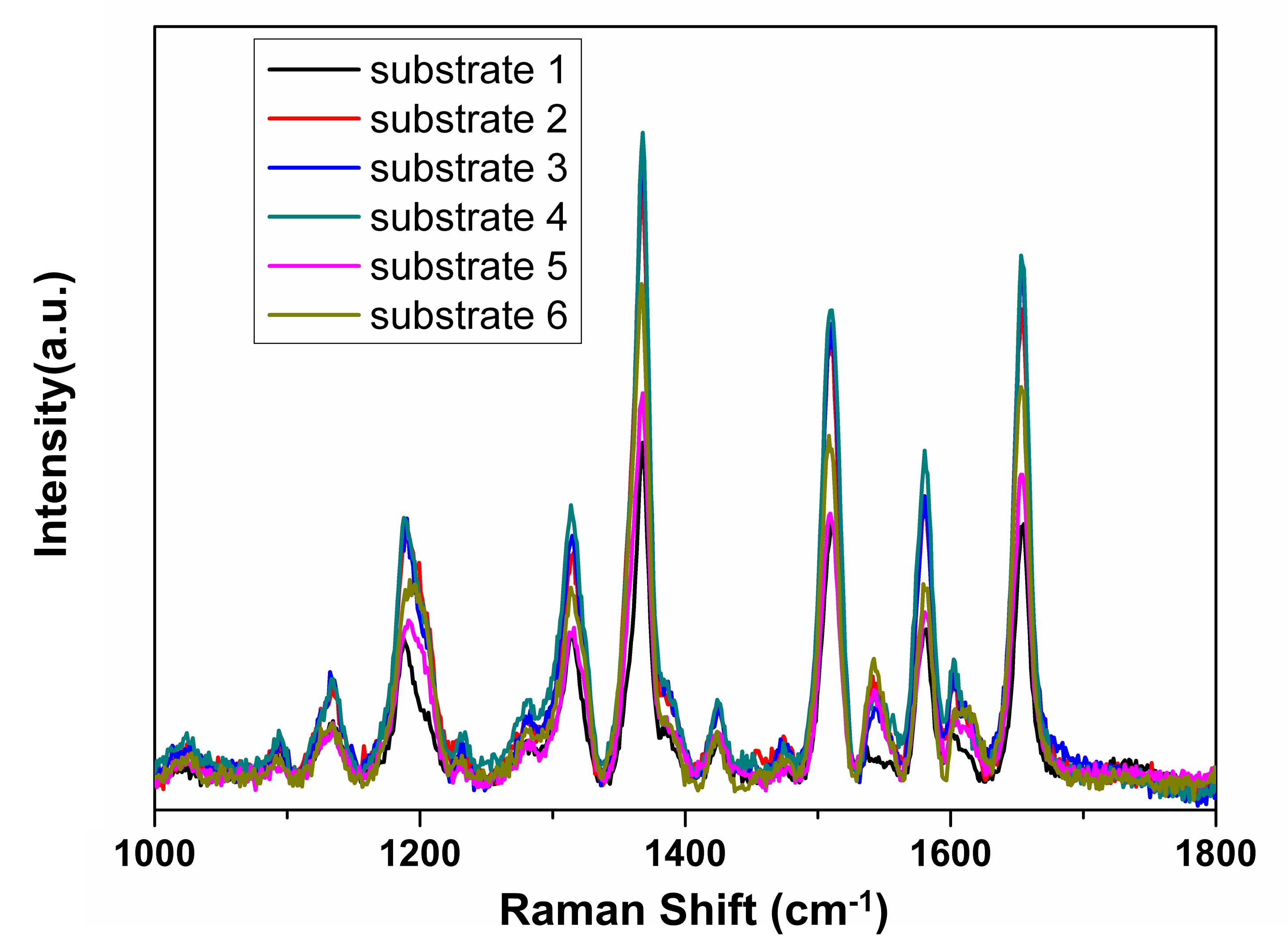


**Fig.2S** The SERS spectra intensity of 6 different substrates produced with the same external current (60 μA).

1. **SERS performance of the front and back of the substrates**

We used 10^-10^ mol·L^-1^ R6G to examine the enhancement effect of the front and back of the silver nanostructures. The silver nanostructure was grown with external current 60 μA. A confocal Raman spectrometer (LabRAM HR Evolution, Horiba Jobin Yvon, France) was used here. The excitation light was a focused Ar^+^ laser (excitation wavelength 514 nm, focal area ~2 μm×2 μm with a 50× objective). The exposure time was 10 s and the detection range was 1000–1800 cm^−1^. The power of the laser was 0.5 mW. The result is shown in Fig.3S. In fact, whether the front or the back of the SERS substrate, the enhancement effect is pretty good. The enhancement effect of front and back of the SERS substrates are of the same order, while the back of SERS substrates is less. So we used the front of the SERS substrates during all the SERS experiments in this study.


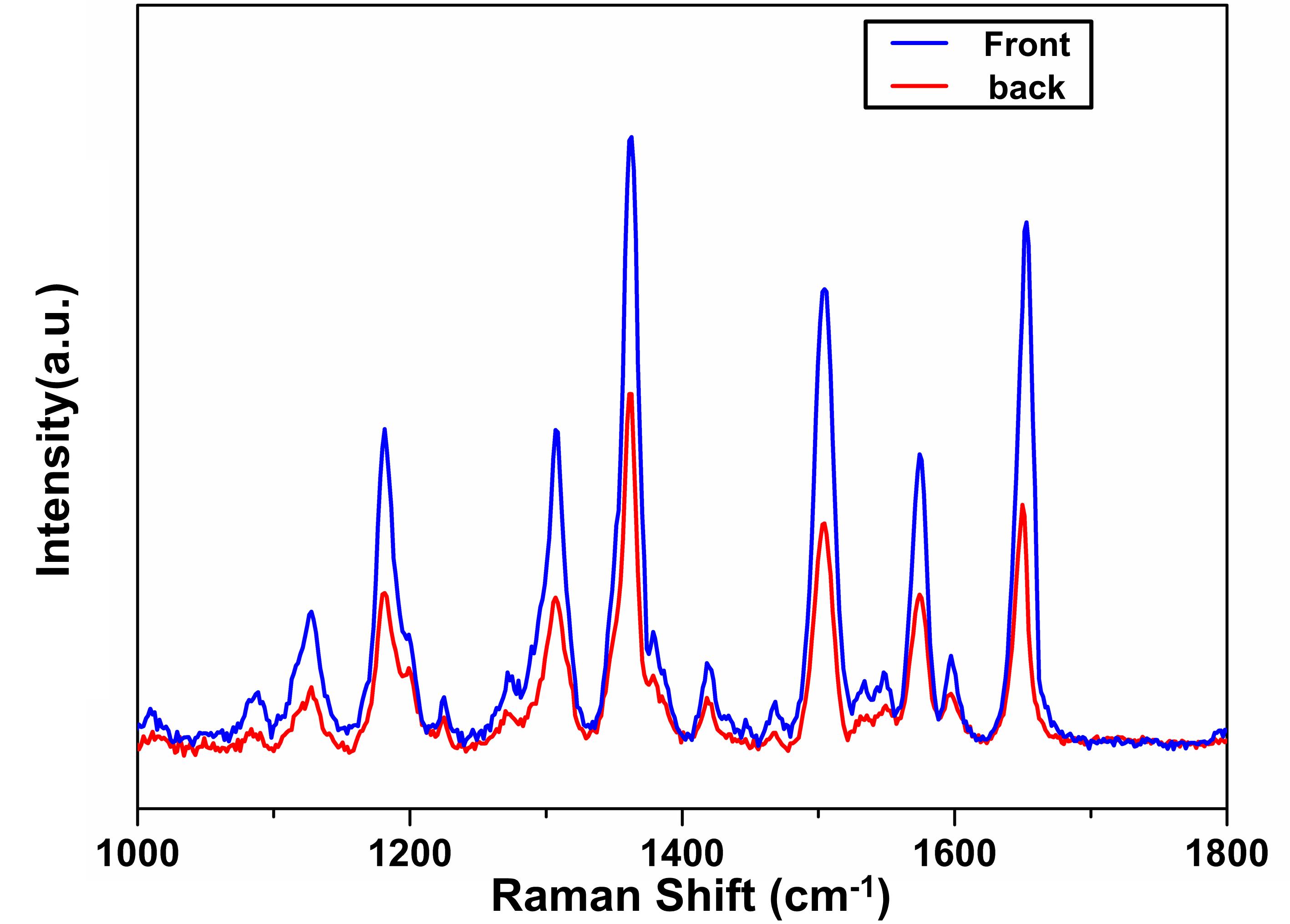


**Fig.3S** The SERS spectra intensity of R6G on the front and back of same silver nanostructure. The blue curve is the SERS spectra of R6G on the front of the silver nanostructure (60 μA) and the red curve is the SERS spectra of R6G on the back of the silver nanostructure (60 μA).

1. **Estimate the enhancement factor of SERS substrates**

We employed the following method to estimate the enhancement factors of the substrates using the real number of molecules probed in the SERS experiments. A confocal Raman microscope (Renishaw InVia, Wotton-under-Edge, UK) for which the laser spot size can be expanded to 500×500 μm with a 5× objective was used to measure the SERS and Raman spectra. We used a pipette with a range of 0.1–1 μL, (Transferpette, Germany) to produce a drop of R6G with a volume of 0.1 μL with an error of less than 2%. The size of the drop was measured with a Spiral-Reading Microscope; the diameter of a drop on the slide was 375 μm, and the diameter of a drop on a SERS substrate was 390 μm. Moreover, the area of a SERS substrate obtained by the solid-state ionics method was large enough to prevent R6G molecules in the drop from leaking off the substrate. As a result, the laser spot can cover and excite all the molecules in the drop, and the number of molecules probed can be calculated from the concentration and volume of the drop. Schematic diagrams outlining sample preparation and Raman/SERS measurement are shown in Fig. 4S(a) and (b).

**
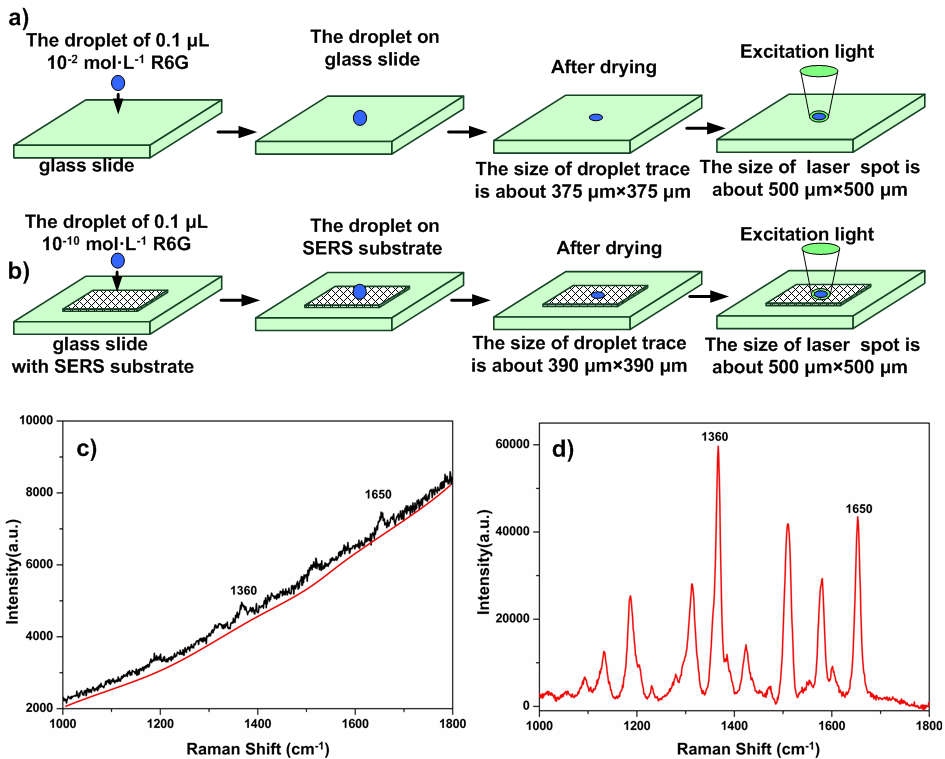
**

**Fig.4S** Schematic diagrams illustrating **(a)** sample preparation and Raman measurement of R6G on a glass slide, and **(b)** sample preparation and SERS measurement of R6G on a SERS substrate. **(c)** Raman spectrum of 0.1 μL of 10^−2^ mol L^−1^ R6G solution on a glass slide. Under the red curve is the fluorescence background. **(d)** SERS spectrum of 0.1 μL of 10^−10^ mol L^−1^ R6G solution on a SERS substrate grown at 60 μA.

We calculated E_F_ of a SERS substrate grown at 60 μA. We used 0.1 μL 10^−2^ mol L^−1^ R6G solution to measure the Raman spectral intensity without SERS substrates. The measurement was carried out with a confocal Raman spectrometer (Renishaw InVia, Wotton-under-Edge, UK). The excitation light was a focused Ar^+^ laser (excitation wavelength 514 nm, focal area 500×500 μm with a 5× objective, laser power 5 mW). The exposure time was 20 s and the detection range was 1000–1800 cm^−1^. The results are shown in Fig. 4S(c). In addition, 0.1 μL of 10^−10^ mol L^−1^ R6G solution was used to measure the SERS intensity with SERS substrates under the same conditions. The resulting spectrum is provided in Fig. 4S(d).

The intensity of peaks was calculated using Origin 8.0. We calculated the intensities of the peaks at 1360 and 1650 cm^−1^. For the peak at 1360 cm^−1^, I_Raman_ = 4182.5, I_SERS_ = 5155950. For that at 1650 cm^−1^, I_Raman_ = 10517.2, I_SERS_ = 993120. N_Raman_/N_SERS_=10^8^. According to the formula:

E_F_= (I_SERS_/I_Raman_)×(N_Raman_/N_SERS_),

for the peak at 1360 cm^−1^, E_F_ = 1.23×10^11^, and for that at 1650 cm^−1^, E_F_ = 9.44×10^10^. Therefore, E_F_ of the substrate grown at 60 μA is about 10^11^. The E_F_ calculated here is the average of the substrate covered with the drop.

**Table S1. Raman bands, assignment and local coordinates for T and R states of Haemoglobin1**

| **Raman band (cm^-1^)** | | **Assignment** | **Local coordinates** |
| --- | --- | --- | --- |
| **T State (Deoxy)** | **R State (Oxy)** |  |
| 674 | 676 | ν_7_ | δ(pyr deform)_sym_ |
| 754 | 755 | ν_15_ | ν(pyr breathing) |
| 973 | 972 | ν_46_ | δ(pyr deform)_asym_ |
| 995 | 1001 | ν_47_ | ν(C_β_C_1_)_asym_ |
| 1082 | 1090 | ν_23_ | ν(C_β_C_1_)_asym_ |
| 1132 | 1135 | ν_22_ | ν(pyr half-ring)_asym_ |
| 1172 | 1171 | ν_30_ | ν(pyr half-ring)_asym_ |
| 1212 |  | ν_5_+ν_18_ | δ(C_m_H) |
| 1301 | 1301 | ν_21_ | δ(C_m_H) |
| 1336 | 1336 | ν_41_ | ν(pyr half-ring)_sym_ |
| 1358 | 1372 | ν_4_  ν_4_ | ν(pyr half-ring)_sym_  ν(pyr half-ring)_sym_ |
| 1397 | 1397 | ν_20_ | ν(pyr quar-ring) |
| 1421 | 1421 | ν_28_ | ν(C_α_C_m_)_sym_ |
| 1471 | 1471 | CH_2_(scissor) | CH_2_(scissor) |
| 1546 | 1547 | ν_11_ | ν(C_α_C_m_) _asym_ |
| 1585 | 1587 | ν_37_ | ν(C_β_C_β_) |
| 1606 |  | ν_19_ | ν(C_α_C_m_)_sym_ |
|  | 1638 | ν_10_ | ν(C_α_C_m_)_sym_ |

**Table S2 Age and gender of RBC samples donors in this work**

| **Donor** | **Age** | **Gender** |
| --- | --- | --- |
| Donor 1 | 78 | F |
| Donor 2 | 82 | F |
| Donor 3 | 85 | F |
| Donor 4 | 68 | F |
| Donor 5 | 73 | M |
| Donor 6 | 77 | M |
| Donor 7 | 82 | M |
| Donor 8 | 60 | M |
| Donor 9 | 60 | M |
| Donor 10 | 65 | F |
| Donor 11 | 68 | F |
| Donor 12 | 75 | M |
| Donor 13 | 20 | F |
| Donor 14 | 22 | F |
| Donor 15 | 26 | F |
| Donor 16 | 28 | M |
| Donor 17 | 28 | M |
| Donor 18 | 19 | M |
| Donor 19 | 20 | F |
| Donor 20 | 18 | F |
| Donor 21 | 19 | M |
| Donor 22 | 20 | M |
| Donor 23 | 26 | M |
| Donor 24 | 27 | M |
| Donor 25 | 29 | F |
| Donor 26 | 29 | F |
| Donor 27 | 22 | F |

**References**

1. Hu, S., Smith, K. M. and Spiro T. G. Assignment of Protoheme Resonance Raman Spectrum by Heme Labelling in Myoglobin. *J. Am. Chem. Soc.* **118**, 8 (1996).
